# Mesotheliomas in Genetically Engineered Mice Unravel Mechanism of Mesothelial Carcinogenesis

**DOI:** 10.3390/ijms19082191

**Published:** 2018-07-27

**Authors:** Didier Jean, Marie-Claude Jaurand

**Affiliations:** 1 Inserm, UMR-1162, Génomique Fonctionnelle des Tumeurs Solides, F-75010 Paris, France; didier.jean@inserm.fr; 2 Université Paris Descartes, Labex Immuno-Oncologie, Sorbonne Paris Cité, F-75000 Paris, France; 3 Institut Universitaire d’Hématologie, Université Paris Diderot, Sorbonne Paris Cité, F-75010 Paris, France; 4 Université Paris 13, Sorbonne Paris Cité, F-93206 Saint-Denis, France

**Keywords:** malignant mesothelioma, mesothelium, mineral fibres, gene mutations, tumor suppressor genes, signalling pathways, carcinogenesis

## Abstract

Malignant mesothelioma (MM), a rare and severe cancer, mainly caused as a result of past-asbestos exposure, is presently a public health concern. Current molecular studies aim to improve the outcome of the disease, providing efficient therapies based on the principles of precision medicine. To model the molecular profile of human malignant mesothelioma, animal models have been developed in rodents, wild type animals and genetically engineered mice harbouring mutations in tumour suppressor genes, especially selecting genes known to be inactivated in human malignant mesothelioma. Animals were either exposed or not exposed to asbestos or to other carcinogenic fibres, to understand the mechanism of action of fibres at the molecular level, and the role of the selected genes in mesothelial carcinogenesis. The aim of the manuscript was to compare mesothelioma models to human malignant mesothelioma and to specify the clue genes playing a role in mesothelial carcinogenesis. Collectively, MM models recapitulate the clinical features of human MM. At least two altered genes are needed to induce malignant mesothelioma in mice. Two pathways regulated by *Cdkn2a* and *Trp53* seem independent key players in mesothelial carcinogenesis. Other genes and pathways appear as bona fide modulators of the neoplastic transformation.

## 1. Introduction

Human malignant mesothelioma (HMM) is a cancer with current poor outcome, which is diagnosed with advanced non-curable disease. HMM has a strong association with asbestos exposure, a natural mineral fibre. The present researches mainly aim to find efficient therapeutics. Many of the current studies focus on target therapy to counteract the physio-pathological mechanisms allowing mesothelioma cells to grow in and invade their microenvironment, and to escape from the immune survey. For that purpose, mesotheliomas are developed in so called “mesothelioma models”, which include orthotopic or heterotopic xenografts of human mesothelioma cell lines and patient-derived xenografts in immunodeficient mice [1,2]. Moreover, experimental mesotheliomas models have been developed for different purposes. Malignant mesotheliomas (MM) models have been generated to understand the carcinogenic mechanism induced by asbestos fibres or to identify the most relevant genes and important signalling pathways associated to mesothelial cell transformation. This aim was developed with both in vitro studies on mammalian cells, including mesothelial cells, and in vivo studies in animals [3]. Efforts have been also made to generate MM in animals treated or not treated with asbestos fibres. More recently, recombinant inbred mouse lines were designed to determine the genetic bases of the disease. In this context, genetically engineered mice (GEM) carrying genes modified to mimic the human disease were chosen and exposed or not to carcinogenic fibres. These experiments allow comparison between mesotheliomas developed in different genetic context and possibly emphasise specific clinical and molecular features.

The application of target therapy needs a deep knowledge of the tumour microenvironment characteristics to permit an appropriate way to suppress tumour cell proliferation, survival, migration, invasiveness and impair the interactions with the microenvironment as final outcome to eradicate the tumour. The different animal models may bring some relevant knowledge of the specific molecular pattern of the tumours and of the disease. In this review, we will discuss the features of mesothelioma induced in animals and to what extent they are close to the HMM.

## 2. Human Malignant Mesothelioma

### 2.1. Human Malignant Pleural Mesothelioma

The clinical and pathological features of pleural MM will be briefly summarised here. Several reviews can be suggested to the reader [4,5,6].

#### 2.1.1. Natural History

The major risk factor for malignant pleural mesothelioma (MPM) is a past exposure to asbestos fibres, and more than 80% of MM are located in the pleura as a result of inhalation exposure. MPM occurs after a long delay, up to 40 years, after the beginning of exposure. However, malignant peritoneal mesothelioma (MPeM) is also found in asbestos-exposed patients, exceptionally in the testis [7]. MPM can be found in populations not exposed for occupational reasons, but showing domestic or environmental exposures [8,9,10,11]

#### 2.1.2. Histological Classification

On the basis of histological morphology, MM is divided into three major histologic types, epithelioid, sarcomatoid, or mixed (biphasic) categories. Epithelioid and sarcomatoid categories have several secondary growth patterns as reported by Hussain et al. [12].

#### 2.1.3. Physiopathology

Mesothelial cells form a monolayer at the surface of the mesothelium. Their cellular morphology is not uniform, depending on the regional location with flattened, intermediate, cuboidal and microvilli-rich mesothelial cells. Mesothelial cells play an important role in maintaining pleural homeostasis [13]. Pleuro-lymphatic communication is made through stomas [14]. In human, stomas open at the mesothelial surface and extend into a lymphatic capillary connected to the submesothelial lymphatics [15]. Inhaled asbestos fibres are deposited in the respiratory airways, reach the lung and are translocated into the pleura. The presence of fibres has been demonstrated both in the human pleura and in animals [16,17].

#### 2.1.4. Molecular Alterations in MPM

Many publications have reported molecular alterations in MPM (see for review [6,18]). They concern copy number alterations (CNAs) of chromosome regions, gene mutations and epigenetic modifications. One recurrent finding is the numerous chromosome rearrangements, with several specific chromosomal gains on 1, 5, 7 and 17 or losses on 1, 3, 4, 6, 9, 13 and 22 [19]. Losses in 3p21, 9p21, 14q and part or whole chromosome 22 were recurrently observed. These loci contain many tumour suppressor genes (TSGs) such as *BAP1*, *CDKN2B*, *CDKN2A*, and *NF2* which are frequently inactivated. Other genes of interest, *LATS2*, *SETD2* and *TP53* are inactivated at a lower extent [20,21,22]. A loss on the chromosome region 14q11.2–q21 was the only difference detected between patients exposed (loss) and not exposed (no loss) to asbestos [23,24]. Gene alterations consisted in base substitution, apuric or apurinic base losses, deletion of one or several exons, or the whole gene. Gene fusions and splice alterations were also described mostly in *NF2*, *BAP1* and *SETD2* genes [22]. So far, no recurrent oncogene was found altered in MM, but an oncogenic hotspot mutation was reported in the promoter of *TERT* in 15% MPM [25]. However amplification of oncogenes such as *PDGFRB*, *MYC* or *VEGFR* could play a role in mesothelial neoplastic transformation [26,27,28].

Investigation of epigenetic changes demonstrated changes in gene methylation, and differential expression in non-coding RNAs such as microRNAs and long non-coding RNAs in comparison with normal cell [29]. It is known that miRNAs interact with the regulation of oncogenes and TSGs and can work either as oncogenes or TSGs [30]. Methylome analyses have shown a variety of methylation profiles in MPM, and an association with asbestos exposure [31]. Analysis of promoter methylation of cell cycle control genes showed that the number of methylated genes was a predictor of asbestos exposure [32]. MiRNome analyses also revealed differential expression between MPM and normal counterparts, between MPM and reactive pleural cells and between histological categories [33,34,35].

#### 2.1.5. Alterations in Regulatory Pathways

Whole genomic and transcriptomic analyses have emphasised the regulatory pathways activated or inactivated in MPM. Hippo and PI3K/AKT/mTOR are deregulated either because of the mutation in critical genes of the pathway and/or inappropriate activation of members of the pathway [36,37,38]. Other regulatory pathways that play a role in development and cell differentiation are reported to be differentially activated in comparison with normal cells, Hedgehog that is associated with the maintenance of cancer stem cells, and Wnt, a pathway, which plays a role in intracellular signal traffic [39,40,41]. Important deregulation of the mitotic spindle assembly checkpoint pathway (MSAC) and microtubule network has been reported in MPM, although no mutation was detected in these genes [42]. Highest levels of expression of genes of the MSAC pathway, notably in sarcomatoid MPM [42].

#### 2.1.6. Molecular Classification of MPM

In addition to histological classification, molecular classification of MPM was performed from trancriptomic analyses. Studying primary MPM cultures and tumour samples by transcriptomic microarray resulted in the definition of two molecular classes (C1 and C2) [43]. Gene mutations were investigated in selected genes *BAP1*, *CDKN2A*, *CDKN2B*, *NF2* and *TP53*. Briefly, *BAP1* alterations were more frequent in C1 and epithelioid MPM were found in both groups, with a worse survival prognosis in the C2 subgroup. Pathway analysis revealed that EMT was differentially regulated between MPM subgroups [43].

In an extensive study, transcriptomes, whole exomes (*n* = 99) and targeted exomes were analysed in MPM tumours [22]. Using RNA-seq data, four molecular subtypes were defined, sarcomatoid, epithelioid, biphasic-epithelioid (biphasic-E) and biphasic-sarcomatoid (biphasic-S). In this study, genes significantly mutated were identified: *BAP1*, *NF2*, *TP53*, *SETD2*, *DDX3X*, *ULK2*, *RYR2*, *CFAP45*, *SETDB1* and *DDX51*, and a multitude of mutations in several genes. These mutations result in the alteration of several signal pathways such as Hippo, mTOR, histone methylation, RNA helicase and p53 pathways. Hippo pathway was altered in all molecular subtypes, mTOR more in biphasic-S. Histone methylation and *BAP1* alteration were more frequent in epithelioid MPM. Six mutation signatures were identified, but none was associated to asbestos exposure [22].

Gene expression was also investigated to differentiate MPM cells and benign mesothelial hyperplasia (MH) using NanoString technologies in tumour tissues [33]. One hundred and seventeen genes were selected. An unsupervised cluster analysis defined two clusters, one composed only of MPM and one only of MH samples. Interestingly, this approach identified already known mesothelioma genes, *BAP1* and *NF2* being downregulated, and *MSLN*, which encodes mesothelin, upregulated in MPM in comparison with MH. In contrast, *CDKN2A* was not statistically deregulated in MPM in comparison with MH [33]. This suggests different roles of these genes in the neoplastic progression of mesothelial cells.

#### 2.1.7. MPM Response to Treatments

There is agreement that globally, MPM survival is dependent on the histological subtype; epithelioid mesothelioma having better prognosis that sarcomatoid mesothelioma. The recent molecular analyses have shown that the outcome of MPM is also related to the molecular group with differential outcome within epithelioid mesothelioma [43].

### 2.2. Malignant Peritoneal Mesothelioma

MPeM also found as a result of asbestos inhalation, is reported as slightly different from MPM. As in MPM, the major histologic types of MPeM as in MPM are found, with the epithelioid type being the most frequent. Histologic variants comprise heterologous (osteosarcomatous, chondrosarcomatous, and rhabdomyosarcomatous) elements and desmoplastic mesothelioma [44]. However, MPeM shows differences with MPM in terms of survival, which is longer than MPM. The main risk factor remains asbestos exposure in about 50% of the cases, lower than in MPM [45].

Genome wide analysis of epithelioid MPeM and MPM showed similarities in CNAs [24]. Overall, regions of copy number gain were more common in MPeM, whereas losses were more common in pleural MPM. Losses occurring in 3p, 9p and 22q genomic regions carrying the TSGs *BAP1*, *CDKN2A* and *NF2*, respectively were seen at a statistically significant higher rate MPM than in MPeM [24]. The authors studied CNAs in groups of different exposures and found different results. Patients with history of medical radiation exposure showed multiple regions of gain, including 1q, 3p, 3q and 5p. Region of losses in 6q, 14q, 17p and 22q and gains 7q, 10p, 10q, 17q were found in tumours from asbestos-exposed patients [24]. Reccurent mutations are also found in similar genes than MPM [46], even if specific alterations were described in subgroup of MPeM such as *ALK* rearrangement [47].

### 2.3. Conclusions on Human Malignant Mesothelioma Biology

HMM appears to have a spectrum of different features. First, MM can grow in the serosa of the pleura, peritoneum, pericardium or tunica vaginalis. The MM tumour morphology is heterogeneous. MM cells in different tumours differ by their physiological and genomic status, and relationship with their microenvironment. Although some physiological and molecular alterations are recurrently found in mesothelioma cells, sometimes at a high rate, given tumours have specific features that need to be known to more precisely define groups of tumours and perform precise therapeutics. In the following, it is discussed to what extent models of MM are close to HMM.

## 3. Models of Malignant Mesothelioma

Mesotheliomas have been developed in rodents by injection of asbestos fibres in wild type (WT) rats or in mice and GEM mice, exposed or non-exposed to asbestos, refractory ceramic fibres (RCF) or carbon nanotubes (CNT).

### 3.1. Spontaneous Mesotheliomas in Wild Type Rodents

Spontaneous mesotheliomas that occur in control or sham cohorts in toxicological studies using rats are rare events. An incidence of 4.3% (7/395) of genital and serosal mesotheliomas, and only one pleural mesothelioma has been reported in male rats, with a variety of morphological patterns [48]. More recently, 0.2–5% mesotheliomas of the tunica vaginalis (MTV) were classified as epithelioid, sarcomatoid of mixed, consistent with the histologic classification in HMM [49]. Spontaneous mesotheliomas were reported in male F344/N rats controls in a summary over 5 decades from 2-year National Toxicology Program carcinogenicity bioassays. The frequency was 0.2–5% MTV [49]. Spontaneous mesothelioma is also rare in mice [50,51,52].

### 3.2. Mesothelioma in Animal Experiments

#### 3.2.1. Asbestos-Induced Mesotheliomas in WT Animals

These studies were carried out mainly in rats, less in mice. The aim was to investigate the carcinogenicity of different types of fibres [53]. Rats were exposed by inhalation, intra-tracheal instillation or intra-serosal administration. Lung tumours and mesotheliomas were observed at different rates, depending on the route of exposure and fibre type [54]. The natural history of mesotheliomas showed similarities with HMM, they occurred after a long delay and ascites developed after exposure via the intra-peritoneal route. Histological analyses reported similar features as found in HMM, but epithelioid is not the most frequent histologic category. For instance, after administration in the pleural cavity of rats, reported histologic types were tubulo-papillary (a category of pleural epithelioid, 8.2%), mixed (74.8%) and spindle (16.9%) MM [55].

Recently, a whole exome sequencing of asbestos-induced murine mesotheliomas (MuMM) was performed in 3 different strains of WT mice stains, BALB/c, CBA and C57BL/6, and 15 MM cell lines were analysed, obtained from 4, 4 and 6 ascites, respectively [56]. In all but one cell line, recurrent genomic changes included homozygous (Hom) loss of *Cdkn2a* (this gene encodes two proteins, p16^Ink4a^ and p19^Arf^) and deletion in *Lats2* and *Setd2*, but no mutation in *Bap1* or *Nƒ2*. Hom loss of *Trp53* was found in one cell line. Mutation signature was principally C to T, as found in HMM, and G to A transitions, but transversions were also found. BALB/c cell lines carried more mutations than the others. Several pathways were affected by mutations such as Wnt, Hedgehog, Notch, mTOR, MAPK and p53 pathways, but not Hippo [56]. These results suggest a unique key role of *Cdkn2a* in murine mesothelial carcinogenesis. Moreover, mesotheliomas arose in the absence of alteration of *Bap1* and *Nƒ2*, as in HMM, consistent with a role of other pathways affected by the genes mutated at low frequency, or epigenetic mechanism.

An epigenetic mechanism of inactivation of *Cdkn2a* locus was suggested to be an initial step of MuMM induction, leading later to allelic deletion of Arf, in WT mice exposed to CNT by intrapleural instillation [57].

#### 3.2.2. Mesothelioma in GEM

##### Spontaneous MuMM

GEM heterozygous (Htz) or homozygous (Hom) in *Nf2*, *Bap1*, *Cdkn2a* (*Ink4a* and/or *Arf*), *Trp53* or *Bap1* genes, either alone or in combination, were generated, based on the knowledge of the TSGs genes playing a role in mesothelial carcinogenesis. GEM in *Rb*, *Tsc1* and *Pten* were also generated despite the absence of mutation in HMM [58,59,60,61]. Table 1 and Table 2 summarize the different studies carried out with GEM.

One MuMM was reported (6%) in *Nƒ2*^KO3/+^ carrying the loss of *Nƒ2* exon 3 [8]. Jongsma et al. [59] injected Ad*Cre* in the pleural cavity of mice carrying conditional mutant alleles in *Nf2*, *Cdkn2a*, *Trp53* or *Rb*, and Htz *Ink4a* mutant [59]. The highest rate of thoracic MuMM was observed in double mutants *Nf2* and *Cdkn2a*, *Trp53* or *Rb* and triple mutants *Nf2*, *Trp53* and *Ink4a*. Mutations in *Cdkn2a*, *Ink4a* or *Trp53* were the most pejorative in term of MuMM incidence. *Rb* inactivation induces the lowest incidence of MuMM. Hom *Nƒ2* enhanced tumours rate in *Rb* mutants [59]. A majority of epithelioid mutants was only found in Hom *Nf2*/Htz *Trp53* mice. Guo et al. [58] injected Ad*Cre* in the peritoneal cavity or in the bladder, in conditional mutants *Trp53* and *Tsc1*. High rate of MuMM was found in double Hom *Trp53/Tsc1* mutants, but none in Htz/Hom mutants. MM were mostly of epithelioid type [58]. Hom *Pten* leads to MuMM with a frequency of 7% in mice, but when coupled with Hom *Trp53*, 56% of mice developed pleural MuMM. The histologies of Hom *Pten* and *Trp53* MuMM were sarcomatoid and biphasic [61].

Three types of Htz *Bap1* mutants were generated in mice, one was knockout in exons 6 and 7 of *Bap1*, and the two others with point mutations identical to germline mutations found in two human families (W and L, respectively) with a *BAP1* cancer syndrome presenting mesothelioma in several family members [60]. Htz germline mutations in *BAP1* predispose to a range of benign and malignant tumours, including mesothelioma. In Htz mice, although numerous types of cancers were developed, mesothelioma was absent or rare (2/93 Htz mice) and developed after a long delay (19 and 29 months). The tumour type with the highest incidence was ovarian sex cord stromal tumours, 38 of 60 (63%) in *Bap1* mutant mice.

Collectively, the results show a differential role of the altered genes. Data from Jongsma et al. [59] suggest a prominent role of *Cdkn2a* and *Trp53*, compared to *Nƒ2*, as mice harbouring Hom *Nƒ2* and either Htz *Cdkn2a* or Htz *Trp53* have longer survival than mice with Hom *Cdkn2a* or *Trp53* and Htz *Nƒ2*. However, Htz *Trp53* in association with Htz or Hom *Tcs1* did not induce MM, contrary to its association with *Nƒ2*, but consistent with a bona fine role of *Nƒ2 in MM* [58,59]. Results also showed that *Bap1* Htz mutations are not sufficient to induce MuMM [60]. All histologic types of mesotheliomas were observed, with a majority of mixed and sarcomatoid types, with the exception of epithelioid type in *Tsc1/Trp53* mice. Despite the different genetic background of mice, these studies underline several key genes for MM, consistent with findings in HMM, and that MM can develop with a variety TGS mutations, and likely with more than one TSG.

##### MuMM in Mice Exposed to Carcinogenic Fibres

Mice harbouring Htz genes *Nƒ2*, *CdKn2a* (*Ink4a* and/or *Arf*), *Trp53* or *Bap1* and their WT counterparts were exposed to carcinogenic fibres administered intra-peritoneally [60,62,63,64,65,66,67,68]. In one study, both *Nƒ2* and *Cdkn2a* were HTz [69]. MuMM arose in both WT and Htz mice, more frequently and with a shorter survival in Htz mice than in their WT counterparts, showing the role of these genes in enhancing sensitivity to fibres. MuMM generally arise after a long delay, often preceded by the occurrence of ascetic fluid. MM were detected several months after exposure, 18 and 27 weeks In Htz *Nƒ2* mice [66,69] and 21 to 37 weeks in Htz *Cdkn2a*, *Ink4a* or *Arf* [63]. Median survivals were around one year or more. From the number of MuMM or lag time after fibre exposure in different genetic situations, it is difficult to establish a hierarchy between genes, because of the variety of protocols between studies (mice strains, dose and schedule of exposure, fibre type). Htz *Trp53* mice were also developed high rate of MuMM when exposed to asbestos or to CNT [68,70].

Additionally, genes other than TSG such as *Asc*, relevant of inflammatory process, was also Hom- or Htz-inactivated in GEM [71]. Inactivation of *Asc* in GEM non-significantly reduced the percentage of mice with MuMM, but the disease-free survival was significantly lowered. These results suggest a role of inflammation in disease progression and the authors showed a relation with IL1b/IL1R signalling [71].

Asbestos induces MuMM in MexTAg transgenic mice that carry a fragment of the Simian Virus 40 (SV40) TAg open reading frame [72]. These MuMM replicate many aspects of MM at the molecular level, but MuMM development was not dependent on *Cdkn2a*, likely attributable to the Tag expression [73].

#### 3.2.3. Mutation Profiles in MuMM of Mice Exposed to Fibres

Genetic alterations have been studied in MM cells cultured from ascitic fluids in fibre-exposed GEM. In MM cells from Htz *Nƒ2* mice, a loss of heterozygosity (LOH) of *Nƒ2* was found in all or a majority of MM cell lines from Htz *Nƒ2* mice, 85% (6/7), 83.5% (10/12) and 100%, respectively [64,65,66]. Inactivation of *Cdkn2a* and *Cdkn2b* was predominant, and resulted from biallelic deletions. Otherwise, co-deletion of *Cdkn2a* (*Ink4a* and *Arf*) and *Cdkn2b* was predominant [64,65,74]. Rates of *Trp53* mutations were less frequent, about 20% as in HMM [64,65,74]. Two cell lines with alteration of *Trp53* were *Cdkn2a* (*Ink4a* and *Arf*) and *Cdkn2b* WT, suggesting two different pathways of carcinogenesis [74]. A role of the hippo pathway is suggested by the activation of Yap/Taz in tissue from asbestos-exposed Htz *Nƒ2* mice, as shown by its nuclear localisation [75].

Altomare et al. [62] reported biallelic inactivation of *Arf* in all cell lines from Htz *Arf* mice, in 3/7 from WT mice, and no deletion of *Ink4a* or *Ink4b* (*Cdkn2b*) in all but one cell line from these mice, and no loss of p53 protein. However, one WT cell line showed loss of *Trp53* and p53 and retention of both *Cdkn2a* and *Cdkn2b.* Most of MM cells from Htz *Arf* mice showed hemizygous loss of *Faf1* and down-regulation of its protein, which regulated TNF-α-mediated NF-κB signalling pathway in these cells. Accordingly, in Htz *Cdkn2a* (*Ink4a* and *Arf*) mice, a biallelic loss of both *Ink4a* and *Arf* was observed, with protein loss of p16^Ink4a^ and p19^ARF^, and in Htz *Ink4a* mice, there was a biallelic inactivation of *Ink4a*, loss of p16^Ink4a^ or p53, and frequent loss p15^Inkba^ and p19^Arf^, but one cell line from Htz *Ink4a* mice expressed p19^Arf^ but did not express p53 [63]. In the three configurations of Htz *Ink4a*, *Arf* or *Cdkn2a* (*Ink4a* and *Arf*), nearly all cell lines expressed Nƒ2 and p53 [63]. The reciprocity between retention and loss of *Cdkn2a* (*Ink4a* and *Arf*) and *Trp53* expression of p53 consistent with an alternative role of the p53 pathway independently of hippo pathway and Ink4a regulation. These results suggest a major role of Arf in a context of fibre exposure and the role of alternative pathways in mesothelial carcinogenesis, as suggested above from the results obtained in Htz *Nƒ2* mice.

Molecular analyses of cell lines from Htz *Bap1* mice showed *Bap1* LOH, but no alteration of *Ink4a*, *Ink4b* and *Arf*, in contrast to WT mice that retain WT Bap1, but were deleted in *Ink4a*, *Ink4b* and *Arf*, suggesting two alternative mechanisms of MM development despite the fact that *CDKN2A* and *BAP1* mutations are not exclusive in HMM [76]. Rb protein was down regulated in cells from Htz *Bap1* mice due to aberrant epigenetic of the *Rb* promoter, suggesting a role of *Bap1* on *Rb* expression [76]. Fifty per cent of MM cell lines from ascites in asbestos-exposed Htz *Trp53* mice had loss of the WT allele. In addition while cell lines with no loss of WT allele were diploid, those with LOH were tetraploid, consistent with a genetic instability related to checkpoint.

In tissues from asbestos-exposed Htz *Nƒ2* mice, Rehrauer et al. [75] reported a higher number of mutations determined by RNA-seq, with an increase in A to G mutations, but not T to C, as compared to sham. This may be due to hydrolytic deamination of adenosine (*Ada*), as *Ada* expression is significantly increased, and linked to an *Adar* downstream activity [75].

## 4. Discussion

Literature data on MM in rodents led us to consider several issues concerning the molecular mechanism of mesothelial cell transformation, and its relationship with exposure to mineral and synthetic fibres. Most studies showed remarkable similarities between human and rodent MM. In both species, MM is a rare spontaneous cancer that is found in the similar sites, pleura, peritoneum and tunica vaginalis. When exposed to carcinogenic fibres, MM occurs after a long delay post-exposure, and all histological categories are observed. From studies carried out in GEM, no single gene predisposes to MM since MuMM are only in fibre-exposed mice, but asbestos is a powerful agent to facilitate the development of MM. MuMM were developed in mice harbouring Htz and Hom inactivation of TSG, or Hom and Hom inactivation.

In WT animals, exposure to fibres induces a significant incidence of MPM or MPeM, depending on the route of administration, respectively, in both rats and mice. The animals were symptomatic, showing ascites after intra-peritoneal administration of fibres. When reported, early MM appeared after several months, and were further detected during the whole life time of the species. In mice, the median survival in animals was about more than one year, except in Hom *Nf2*/*Trp53* and Hom *Nƒ2*/*Trp53*/*Ink4a*. The survival was lower in asbestos-exposed GEM mice.

Although no precise quantitative data in the distribution of histological categories are given, the epithelioid type is not the most frequent in WT rodents and in GEM. In GEM the most frequent categories are sarcomatoid or mixed MPeM. In contrast, the epithelioid type is the most frequent human MPeM. However, a prevalence of epithelioid MPeM was reported in GEM Hom *Trp53*/*Tsc1* not exposed to asbestos [58], and in both WT and Htz *Arf* asbestos-exposed mice [62].

Investigations of spontaneous MM in GEM harbouring co-mutations in TSG showed that two genes, *Cdkn2a* and *Trp53* are predominant for MM development, as biallelic inactivation generates the highest rate of MM [58,59]. This is found despite the biallelic inactivation of *Nƒ2*, suggesting a cooperative but not predominant role of this gene [59]. Accordingly, in asbestos-exposed Htz *Nƒ2* mice, *Nƒ2* LOH is associated to loss of *Cdkn2a* and/or *Cdkn2b*. A key role of *Cdkn2a* and *Trp53* is also seen when using cell cultured from ascites fluids from Htz *Cdkn2a*, *Ink4a* and *Arf* GEM. Among genes encoded at the *Ink4* locus (*Ink4a*, *Ink4b* and *Arf*), *Trp53* biallelic inactivation is an alternative mechanism to carcinogenesis via genes inactivated at the *Ink4* locus. Of note, TP53 mutations are found in 11% of HMM (Cosmic database v85, https://cancer.sanger.ac.uk). Interestingly, in Htz *Bap1* mice, *Cdkn2a* or *Cdkn2a*b are not inactivated in MM, in contrast to MM with WT Bap1 where both genes are lost, but *Rb* down regulation was evidenced [76]. Independently of the inactivation of TSG already known to be involved in MM, mutations in genes involved in other regulatory pathways act as complementary mechanism accounting for mesothelial carcinogenesis.

As a whole, these studies brought information on the molecular changes in MM. A few genes are key players in the carcinogenic process. Others are bona fide modulators, which may be requested to favour the progression of the tumour, due to their involvement in signal or metabolic pathways. The diversity of mutated genes, the complex combination of altered genes, and the variety of associated deregulated pathways, lead to the heterogeneity of the tumour molecular profiles and is in agreement with the inter-tumour heterogeneity observed in HMM.

## 5. Conclusions

The data on asbestos-exposed mice do not bring significant information on the mechanism of genotoxicity of asbestos fibres. A better identification of the mutation signatures, characterisation of deleted regions and break points localisation and epigenetic changes, in both MM tumours and MM cell lines could help understanding the mechanism genome damage [77]. Inflammation is thought to act as a contributor, but it is not known whether it is the driving force for DNA damaging at lower doses than required in experiments [78]. Events entailing gene deletion and rearrangements should be considered. The contribution of gene methylation is not enough documented, but *Rb* is regulated by DNA methylation in Htz *Bap1* mice [76]. Jongsma et al. [59] reported that epigenetic inactivation of *Ink4a*, although enhancing the malignancy, does not contribute to the development of pleural MuMM in Htz *Nƒ2*/*Trp53*, in agreement with the evidence of deletions of this gene demonstrated in several studies [59]. Nevertheless, hypermethylation of *Cdkn2a* locus preceding allelic *Arf* deletion was suggested to be a mesothelial carcinogenesis step in pleura of mice exposed to CNT [57]. These studies have emphasised the diversity of the molecular events entailing the development of MM in experimental animals, and their consistency with the molecular status of HMM, in term of key genes and pathways, and potent modulators of tumour progression.

## Figures and Tables

**Table 1:** Induction of murine mesotheliomas (MuMM) in genetically engineered mice (GEM) (Injection of Ad*Cre* in GEM ^1^).

Gene(s) Affected	Gene(s) Status	MuMM %	Epi ^2^ %	Sarco ^2^ %	Mixed %	Survival ^3^ Weeks	Reference
*Nf2* *Ink4a/Arf*	Htz Hom	34 ^4^	28.6	21.4	50	58 ^5^	[59]
*Nf2* *Ink4a/Arf*	Hom Htz	34.6 ^4^	22.2	27.8	50	71 ^5^	[59]
*Nf2* *Ink4a/Arf*	Hom Hom	79 ^4^	2.2	68.9	28.9	31 ^5^	[59]
*Nf2* *Rb*	Htz Hom	5.9 ^4^	0	Primarily sarco	Some mixed	ND ^7^	[59]
*Nf2* *Rb*	Hom Htz	13.3 ^4^	0	Primarily sarco	Some mixed	ND	[59]
*Nf2* *Rb*	Hom Hom	26.3 ^4^	0	Primarily sarco	Some mixed	ND	[59]
*Nf2* *Rb*	Htz Hom	6.75 ^6^	0	Primarily sarco	Some mixed	ND	[59]
*Nf2* *Rb*	Hom Htz	13.3 ^6^	0	Primarily sarco	Some mixed	ND	[59]
*Nf2* *Rb*	Hom Hom	20 ^6^	0	Primarily sarco	Some mixed	ND	[59]
*Nf2* *Trp53*	Htz Hom	59 ^4^	25	25	50	29 ^5^	[59]
*Nf2* *Trp53*	Hom Htz	25 ^4^	60	40	0	86 ^5^	[59]
*Nf2* *Trp53*	Hom Hom	82 ^4^	15.5	46.7	37.8	19 ^5^	[59]
*Nf2* *Trp53* *Ink4a*	Hom Hom Htz	93.7 ^4^	0	40	60	ND	[59]
*Nf2* *Trp53* *Ink4a*	Hom Hom Hom	91.1 ^4^	0	76.6	23.4	11	[59]
*Tsc1* *Tp53*	Hom Hom	85 ^6^	Mostly			37	[58]
*Tsc1* *Tp53*	Hom WT	0 ^6^	NA ^7^			>57	[58]
*Tsc1* *Tp53*	WT Hom	0 ^6^	NA			>57	[58]
*Tsc1* *Tp53*	WT WT	0 ^6^	NA			>57	[58]
*Tsc1* *Tp53*	Hom Hom	73 ^8^	Mostly			44	[58]
*Tsc1* *Tp53*	Hom WT	0 ^8^	NA			>57	[58]
*Tsc1* *Tp53*	WT Hom	0 ^8^	NA			>57	[58]
*Tsc1* *Tp53*	WT WT	0 ^8^	NA			>57	[58]

^1^ Strain of mice: FVB/N [59]; Hybrids [58]; ^2^ Epi.: Epithelioid; Sarco.: Sarcomatoid; ^3^ Median survival of the series; ^4^ After intrathoracic injection of Ad*Cre*; ^5^ Mice with thoracic tumours; ^6^ After intraperitoneal injection of Ad*Cre*; ^7^ ND: Not done; NA: Not applicable; ^8^ After injection of Ad*Cre* in the bladder.

**Table 2:** Induction of MuMM in GEM (Induction of MuMM by injection of fibres).

Mice Strain	Gene(s) Affected	Gene(s) Status	Treatment	MuMM %	Epi ^2^ %	Sarco ^2^ %	Mixed %	Survival ^3^ Weeks	Reference
FVB/N	*Nf2*	Htz	Asbestos	47	30.4 Htz + WT	65.2 Htz + WT	4.3 Htz + WT		[66]
FVB/N	None	WT	Asbestos	15	30.4 Htz + WT	65.2 Htz + WT	4.3 Htz + WT		[66]
FVB/N	*Nf2*	Htz	Saline	0	NA	NA	NA		[66]
FVB/N	None	WT	Saline	0	NA	NA	NA		[66]
129Sv/Jae	*Nf2*	Htz	Asbestos	85	6.25	18.75	75	43	[64]
129Sv/Jae	None	WT	Asbestos	59	31	27.6	41.4	52	[64]
FVB/N	*Nf2*	Htz	RCF	55	27	38.4	34.6	68	[65]
FVB/N	None	WT	RCF	7.1	0	0	100	80	[65]
C57/Bl6	*Nf2*	Htz	Asbestos	10	ND	ND	ND	ND	[75]
C57/Bl6	*Arf*	Htz	Asbestos	96.2	68	12	20	42	[62]
C57/Bl6	None	WT	Asbestos	81.5	68.2	18.2	13.6	56	[62]
Hybrids	*Ink4a/Arf*	Htz	Asbestos	88	Occasional	Prevalent	Occasional	29.6	[63]
Hybrids	*Ink4a/Arf*	Htz	TiO_2_	0	NA	NA	NA	NA	[63]
Hybrids	*Ink4a*	Htz	Asbestos	66	Occasional	Prevalent	Occasional	34.6	[63]
Hybrids	*Arf*	Htz	Asbestos	65	Occasional	Prevalent	Occasional	38	[63]
Hybrids	None	WT	Asbestos	50	Occasional	Prevalent	Occasional	49.4	[63]
Hybrids	*Nf2*	Htz	Asbestos	ND	ND	ND	ND	38	[69]
Hybrids	*Nf2Ink4a/Arf*	HtzHtz	Asbestos	ND	ND	Most sarcomatous	ND	24	[69]
Hybrids	None	WT	Asbestos	ND	ND	ND	ND	45	[69]
129/Sv on a 75% C57/Bl6 background	*Trp53*	Htz	Asbestos	76 (after 44 weeks)	ND	ND	ND		[68]
129/Sv on a 75% C57/Bl6 background	*Trp53*	Hom	Asbestos	ND	ND	ND	ND		[68]
129/Sv on a 75% C57/Bl6 background	None	WT	Asbestos	32 (after 67 weeks)	ND	ND	ND		[68]
FVB	*Bap1*	Htz	Asbestos	73	ND	ND	ND	43	[76]
FVB	None	WT	Asbestos	32	ND	ND	ND	55	[76]
FVB	*Bap1*	Htz (L)	Asbestos	71	ND	ND	ND	46	[60]
FVB	*Bap1*	Htz (W)	Asbestos	74	ND	ND	ND	48	[60]
FVB	None	WT	Asbestos	35	ND	ND	ND	60	[60]
C57BL/6	*Bap1*	Htz	Asbestos low dose	36		all or part		57	[67]
C57BL/6	None	WT	Asbestos low dose	10		all or part		57	[67]
C57BL/6	None	WT	Saline	0		NA		NA	[67]
C57BL/6	*Bap1*	Htz	Asbestos std dose	60		all or part		39	[67]
C57BL/6	*Bap1*	WT	Asbestos std dose	28		all or part		57	[67]
C57BL/6	*Asc*	Hom	Asbestos	55	0	75	25	66.2	[71]
C57BL/6	*Asc*	Htz	Asbestos	65	0	68	32	69.4	[71]
C57BL/6	None	WT	Asbestos	80	0	67	33	OK	[71]

^2^ Epi.: Epithelioid; Sarco.: Sarcomatoid; ^3^ Median survival of the series.

## Abbreviations

CNT	Carbon Nanotubes
GEM	Genetically Engineered Mice
HMM	Human Malignant Mesothelioma
MM	Malignant Mesothelioma
MPeM	Malignant Peritoneal Mesothelioma
MuMM	Murine Malignant Mesothelioma
